# Dichloridobis(5-heptyl-1,3,4-thia­diazol-2-amine-κ*N* ^3^)zinc(II)

**DOI:** 10.1107/S1600536809032073

**Published:** 2009-08-19

**Authors:** Peng Wang, Rong Wan, Bin Wang, Feng Han, Yao Wang

**Affiliations:** a Department of Applied Chemistry, College of Science, Nanjing University of Technology, No. 5 Xinmofan Road, Nanjing, Nanjing 210009, People’s Republic of China

## Abstract

In the title compound, [ZnCl_2_(C_9_H_17_N_3_S)_2_], the Zn^II^ atom is four-coordinated by two N atoms from two 5-heptyl-1,3,4-thia­diazol-2-amine ligands and two Cl atoms in a distorted tetra­hedral geometry. The thia­diazole rings are oriented at a dihedral angle of 84.87 (4)°. Intra­molecular N—H⋯Cl inter­actions result in the formation of two six-membered rings having envelope and planar conformations. In the crystal structure, inter­molecular N—H⋯N and N—H⋯Cl inter­actions link the mol­ecules into a three-dimensional network. π–π contacts between thia­diazole rings [centroid–centroid distance = 3.602 (1) Å] may further stabilize the structure.

## Related literature

For general background to thia­diazo­les and their derivatives, see: Alzuet *et al.* (2003 ▶); Shen *et al.* (2004 ▶).
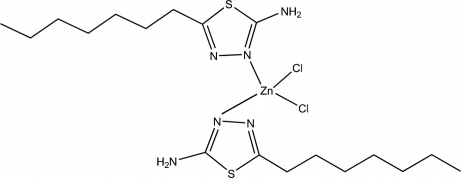


## Experimental

### 

#### Crystal data

[ZnCl_2_(C_9_H_17_N_3_S)_2_]
*M* *_r_* = 534.94
Triclinic, 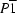

*a* = 8.1750 (16) Å
*b* = 11.663 (2) Å
*c* = 14.666 (3) Å
α = 73.150 (17)°
β = 77.83 (2)°
γ = 88.81 (3)°
*V* = 1307.0 (5) Å^3^
*Z* = 2
Mo *K*α radiation
μ = 1.32 mm^−1^
*T* = 294 K
0.30 × 0.20 × 0.10 mm

#### Data collection

Enraf–Nonius CAD-4 diffractometer
Absorption correction: ψ scan (North *et al.*, 1968 ▶) *T*_min_ = 0.693, *T*_max_ = 0.879
5096 measured reflections
4734 independent reflections
3303 reflections with *I* > 2σ(*I*)
*R*_int_ = 0.054
3 standard reflections frequency: 120 min intensity decay: 1%

#### Refinement

*R*[*F* ^2^ > 2σ(*F* ^2^)] = 0.062
*wR*(*F* ^2^) = 0.177
*S* = 1.02
4734 reflections
256 parameters
H-atom parameters constrained
Δρ_max_ = 1.17 e Å^−3^
Δρ_min_ = −1.27 e Å^−3^

### 

Data collection: *CAD-4 Software* (Enraf–Nonius, 1989 ▶); cell refinement: *CAD-4 Software*; data reduction: *XCAD4* (Harms & Wocadlo, 1995 ▶); program(s) used to solve structure: *SHELXS97* (Sheldrick, 2008 ▶); program(s) used to refine structure: *SHELXL97* (Sheldrick, 2008 ▶); molecular graphics: *SHELXTL* (Sheldrick, 2008 ▶); software used to prepare material for publication: *SHELXL97*.

## Supplementary Material

Crystal structure: contains datablocks global, I. DOI: 10.1107/S1600536809032073/hk2748sup1.cif

Structure factors: contains datablocks I. DOI: 10.1107/S1600536809032073/hk2748Isup2.hkl

Additional supplementary materials:  crystallographic information; 3D view; checkCIF report

## Figures and Tables

**Table:** 

Zn—Cl1	2.2283 (16)
Zn—Cl2	2.2626 (17)
Zn—N1	2.037 (4)
Zn—N4	2.026 (4)

**Table:** 

Cl1—Zn—Cl2	114.97 (7)
N1—Zn—Cl1	109.00 (12)
N1—Zn—Cl2	106.06 (13)
N4—Zn—Cl1	112.65 (12)
N4—Zn—Cl2	108.05 (13)
N4—Zn—N1	105.49 (16)

**Table 2:** Hydrogen-bond geometry (Å, °)

*D*—H⋯*A*	*D*—H	H⋯*A*	*D*⋯*A*	*D*—H⋯*A*
N3—H3*A*⋯Cl1	0.86	2.58	3.374 (5)	154
N3—H3*B*⋯Cl2^i^	0.86	2.77	3.503 (5)	144
N6—H6*A*⋯Cl2	0.86	2.49	3.289 (5)	155
N6—H6*B*⋯N2^ii^	0.86	2.19	3.018 (3)	163

Symmetry codes: (i) 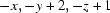; (ii) 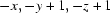.

**Fig. 1.**
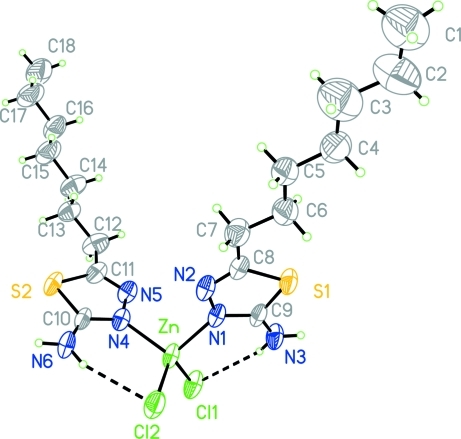
The molecular structure of the title molecule, with the atom-numbering scheme. Displacement ellipsoids are drawn at the 50% probability level. Hydrogen bonds are shown as dashed lines.

**Fig. 2.**
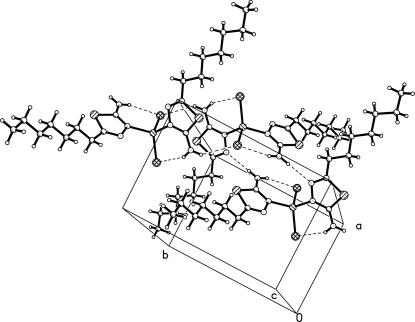
A partial packing diagram. Hydrogen bonds are shown as dashed lines.

## supplementary crystallographic information

### Comment

As a series of superior ligands, thiadiazoles and their derivatives can coordinate to many metal ions with N or S atoms of the five-membered ring. In particular N,N'-linkage ligands, such as 1,3,4-thiadiazoles, are very versatile compounds that are able to bridge a wide range of inter-metallic separations through two close adjacent N donors (Alzuet *et al.*,  2003). These complexes have received considerable attention in past few years, due to their certain antibacterial and antifungal activities (Shen *et al.*,  2004).

In the molecule of the title compound, (Fig. 1), Zn^II^ atom is four-coordinated by two N atoms from two 5-heptyl-[1,3,4]thiadiazol-2-ylamine ligands and two Cl atoms in a distorted tetrahedral geometry (Table 1). Rings A (S1/N1/N2/C8/C9) and B (S2/N4/N5/C10/C11) are, of course, planar and they are oriented at a dihedral angle of A/B = 84.87 (4)°. The intramolecular N-H···Cl interactions (Table 2) result in the formations of two six-membered rings C (Zn/Cl1/N1/N3/C9/H3A) and D (Zn/Cl2/N4/N6/C10/H6A). Ring C adopts envelope conformation with atom Zn displaced by -0.318 (3) Å from the plane of the other ring atoms, while ring D is planar and it is oriented with respect to the adjacent ring B at a dihedral angle of B/D = 1.08 (4)°. So, they are almost coplanar.

In the crystal structure, intermolecular N-H···N and N-H···Cl interactions (Table 2) link the molecules into a three-dimensional network (Fig. 2), in which they may be effective in the stabilization of the structure. The π–π contact between the thiadiazole rings, Cg2—Cg2^i^, [symmetry code: (i) 1 - x, 1 - y, 1 - z, where Cg2 is centroid of the ring B (S2/N4/N5/C10/C11)] may further stabilize the structure, with centroid-centroid distance of 3.602 (1) Å.

### Experimental

For the preparation of the title compound, ZnCl_2_ ethanol solution (0.5 mmol) was slowly added into a solution of 5-heptyl-[1,3,4]thiadiazol-2-ylamine (1 mmol) in ethanol (20 ml), and then heated under reflux for 2 h. The reaction mixture was left to cool to room temperature, filtrated, and the solid was recrystallized from ethanol to give the title compound (m.p. 426 K). Crystals suitable for X-ray analysis were obtained by slow evaporation of an acetone solution.

### Refinement

H atoms were positioned geometrically, with N-H = O.86 Å (for NH_2_) and C-H = 0.97 and 0.96 Å for methylene and methyl H, respectively, and constrained to ride on their parent atoms, with U_iso_(H) = xU_eq_(C,N), where x = 1.5 for methyl H and x = 1.2 for all other H atoms.

### Crystal data

**Table:**

[ZnCl_2_(C_9_H_17_N_3_S)_2_]	*Z* = 2
*M**_r_* = 534.94	*F*(000) = 560
Triclinic, *P*1	*D*_x_ = 1.359 Mg m^−^^3^
Hall symbol: -P 1	Melting point: 426 K
*a* = 8.1750 (16) Å	Mo *K*α radiation, λ = 0.71073 Å
*b* = 11.663 (2) Å	Cell parameters from 25 reflections
*c* = 14.666 (3) Å	θ = 10–14°
α = 73.150 (17)°	µ = 1.32 mm^−^^1^
β = 77.83 (2)°	*T* = 294 K
γ = 88.81 (3)°	Block, yellow
*V* = 1307.0 (5) Å^3^	0.30 × 0.20 × 0.10 mm

### Data collection

**Table:**

Enraf–Nonius CAD-4 diffractometer	3303 reflections with *I* > 2σ(*I*)
Radiation source: fine-focus sealed tube	*R*_int_ = 0.054
graphite	θ_max_ = 25.3°, θ_min_ = 1.5°
ω/2θ scans	*h* = 0→9
Absorption correction: ψ scan (North *et al.*, 1968)	*k* = −14→14
*T*_min_ = 0.693, *T*_max_ = 0.879	*l* = −17→17
5096 measured reflections	3 standard reflections every 120 min
4734 independent reflections	intensity decay: 1%

### Refinement

**Table:**

Refinement on *F*^2^	Secondary atom site location: difference Fourier map
Least-squares matrix: full	Hydrogen site location: inferred from neighbouring sites
*R*[*F*^2^ > 2σ(*F*^2^)] = 0.062	H-atom parameters constrained
*wR*(*F*^2^) = 0.177	*w* = 1/[σ^2^(*F*_o_^2^) + (0.1*P*)^2^ + 0.5*P*] where *P* = (*F*_o_^2^ + 2*F*_c_^2^)/3
*S* = 1.02	(Δ/σ)_max_ < 0.001
4734 reflections	Δρ_max_ = 1.17 e Å^−^^3^
256 parameters	Δρ_min_ = −1.27 e Å^−^^3^
Primary atom site location: structure-invariant direct methods	

### Special details

**Table:**

Geometry. All e.s.d.'s (except the e.s.d. in the dihedral angle between two l.s. planes) are estimated using the full covariance matrix. The cell e.s.d.'s are taken into account individually in the estimation of e.s.d.'s in distances, angles and torsion angles; correlations between e.s.d.'s in cell parameters are only used when they are defined by crystal symmetry. An approximate (isotropic) treatment of cell e.s.d.'s is used for estimating e.s.d.'s involving l.s. planes.
Refinement. Refinement of *F*^2^ against ALL reflections. The weighted *R*-factor *wR* and goodness of fit *S* are based on *F*^2^, conventional *R*-factors *R* are based on *F*, with *F* set to zero for negative *F*^2^. The threshold expression of *F*^2^ > σ(*F*^2^) is used only for calculating *R*-factors(gt) *etc*. and is not relevant to the choice of reflections for refinement. *R*-factors based on *F*^2^ are statistically about twice as large as those based on *F*, and *R*- factors based on ALL data will be even larger.

### Fractional atomic coordinates and isotropic or equivalent isotropic displacement parameters (Å^2^)

**Table:**

	*x*	*y*	*z*	*U*_iso_*/*U*_eq_	
Zn	0.20470 (7)	0.74792 (5)	0.52670 (5)	0.0475 (2)	
Cl1	0.42266 (17)	0.84281 (12)	0.54632 (13)	0.0631 (4)	
Cl2	−0.00212 (19)	0.68349 (12)	0.66162 (11)	0.0612 (4)	
S1	−0.04746 (18)	1.03689 (11)	0.32771 (11)	0.0531 (4)	
S2	0.32968 (18)	0.40780 (12)	0.43889 (11)	0.0548 (4)	
N1	0.0979 (5)	0.8621 (3)	0.4239 (3)	0.0457 (10)	
N2	−0.0286 (5)	0.8113 (4)	0.3960 (3)	0.0487 (11)	
N3	0.2131 (6)	1.0514 (4)	0.4083 (3)	0.0555 (12)	
H3A	0.2872	1.0198	0.4407	0.067*	
H3B	0.2106	1.1281	0.3862	0.067*	
N4	0.2722 (5)	0.6065 (3)	0.4752 (3)	0.0451 (10)	
N5	0.3971 (5)	0.6318 (4)	0.3912 (3)	0.0523 (11)	
N6	0.1068 (6)	0.4464 (4)	0.5882 (3)	0.0559 (12)	
H6A	0.0580	0.4913	0.6222	0.067*	
H6B	0.0802	0.3710	0.6061	0.067*	
C1	−0.8999 (12)	1.1163 (10)	0.0104 (8)	0.136 (3)	
H1A	−0.9142	1.1855	−0.0415	0.204*	
H1B	−0.9989	1.1008	0.0615	0.204*	
H1C	−0.8808	1.0481	−0.0141	0.204*	
C2	−0.7616 (13)	1.1374 (11)	0.0474 (8)	0.139 (4)	
H2B	−0.7931	1.1902	0.0879	0.167*	
H2C	−0.6715	1.1776	−0.0065	0.167*	
C3	−0.6975 (13)	1.0203 (10)	0.1083 (8)	0.136 (3)	
H3C	−0.7895	0.9770	0.1593	0.163*	
H3D	−0.6578	0.9699	0.0666	0.163*	
C4	−0.5561 (9)	1.0448 (6)	0.1542 (5)	0.0778 (19)	
H4A	−0.4670	1.0909	0.1026	0.093*	
H4B	−0.5979	1.0943	0.1963	0.093*	
C5	−0.4821 (8)	0.9362 (5)	0.2128 (5)	0.0627 (15)	
H5B	−0.5695	0.8910	0.2661	0.075*	
H5C	−0.4418	0.8853	0.1717	0.075*	
C6	−0.3420 (8)	0.9659 (5)	0.2535 (5)	0.0655 (16)	
H6C	−0.3843	1.0133	0.2972	0.079*	
H6D	−0.2580	1.0152	0.2003	0.079*	
C7	−0.2584 (7)	0.8582 (5)	0.3084 (5)	0.0642 (16)	
H7A	−0.3416	0.8100	0.3627	0.077*	
H7B	−0.2189	0.8095	0.2653	0.077*	
C8	−0.1135 (7)	0.8886 (4)	0.3469 (4)	0.0468 (12)	
C9	0.1032 (6)	0.9821 (4)	0.3923 (4)	0.0443 (12)	
C10	0.2237 (6)	0.4937 (4)	0.5079 (4)	0.0419 (11)	
C11	0.4388 (7)	0.5392 (5)	0.3641 (4)	0.0522 (13)	
C12	0.5716 (8)	0.5407 (7)	0.2756 (5)	0.0735 (19)	
H12A	0.5794	0.6204	0.2299	0.088*	
H12B	0.6781	0.5271	0.2953	0.088*	
C13	0.5476 (8)	0.4520 (6)	0.2231 (4)	0.0669 (17)	
H13A	0.4385	0.4615	0.2063	0.080*	
H13B	0.5493	0.3716	0.2663	0.080*	
C14	0.6806 (9)	0.4664 (7)	0.1313 (5)	0.082 (2)	
H14A	0.7892	0.4545	0.1486	0.098*	
H14B	0.6813	0.5478	0.0893	0.098*	
C15	0.6560 (10)	0.3794 (7)	0.0742 (5)	0.088 (2)	
H15A	0.6645	0.2982	0.1144	0.106*	
H15B	0.5438	0.3866	0.0617	0.106*	
C16	0.7804 (9)	0.3996 (7)	−0.0220 (5)	0.083 (2)	
H16A	0.7723	0.4809	−0.0623	0.100*	
H16B	0.8928	0.3920	−0.0097	0.100*	
C17	0.7537 (11)	0.3134 (8)	−0.0773 (6)	0.101 (3)	
H17A	0.6398	0.3183	−0.0871	0.121*	
H17B	0.7672	0.2324	−0.0384	0.121*	
C18	0.8756 (11)	0.3384 (8)	−0.1771 (6)	0.112 (3)	
H18A	0.8566	0.2788	−0.2076	0.168*	
H18B	0.9888	0.3357	−0.1682	0.168*	
H18C	0.8574	0.4164	−0.2178	0.168*	

### Atomic displacement parameters (Å^2^)

**Table:**

	*U*^11^	*U*^22^	*U*^33^	*U*^12^	*U*^13^	*U*^23^
Zn	0.0495 (4)	0.0276 (3)	0.0700 (5)	0.0037 (2)	−0.0124 (3)	−0.0214 (3)
Cl1	0.0533 (8)	0.0424 (7)	0.1056 (12)	0.0043 (6)	−0.0236 (8)	−0.0354 (8)
Cl2	0.0691 (9)	0.0387 (7)	0.0739 (10)	−0.0038 (6)	−0.0015 (7)	−0.0231 (7)
S1	0.0633 (9)	0.0291 (6)	0.0693 (10)	0.0029 (6)	−0.0160 (7)	−0.0168 (6)
S2	0.0619 (9)	0.0385 (7)	0.0755 (10)	0.0096 (6)	−0.0170 (7)	−0.0332 (7)
N1	0.051 (2)	0.031 (2)	0.059 (3)	0.0020 (18)	−0.009 (2)	−0.0206 (19)
N2	0.051 (3)	0.032 (2)	0.065 (3)	−0.0010 (19)	−0.009 (2)	−0.019 (2)
N3	0.061 (3)	0.030 (2)	0.078 (3)	0.000 (2)	−0.020 (2)	−0.016 (2)
N4	0.046 (2)	0.034 (2)	0.059 (3)	0.0023 (18)	−0.012 (2)	−0.019 (2)
N5	0.055 (3)	0.043 (3)	0.063 (3)	−0.002 (2)	−0.011 (2)	−0.022 (2)
N6	0.067 (3)	0.028 (2)	0.075 (3)	0.003 (2)	−0.013 (3)	−0.021 (2)
C1	0.126 (4)	0.151 (5)	0.134 (5)	0.016 (4)	−0.042 (4)	−0.035 (4)
C2	0.137 (7)	0.161 (7)	0.126 (7)	0.010 (6)	−0.061 (6)	−0.026 (6)
C3	0.126 (4)	0.151 (5)	0.134 (5)	0.016 (4)	−0.042 (4)	−0.035 (4)
C4	0.089 (4)	0.070 (4)	0.075 (4)	0.001 (3)	−0.013 (3)	−0.026 (3)
C5	0.068 (4)	0.056 (4)	0.067 (4)	0.003 (3)	−0.019 (3)	−0.019 (3)
C6	0.074 (4)	0.054 (3)	0.072 (4)	0.001 (3)	−0.023 (3)	−0.018 (3)
C7	0.064 (4)	0.054 (3)	0.080 (4)	0.002 (3)	−0.021 (3)	−0.024 (3)
C8	0.051 (3)	0.037 (3)	0.056 (3)	0.003 (2)	−0.007 (3)	−0.021 (2)
C9	0.050 (3)	0.029 (2)	0.054 (3)	0.002 (2)	−0.001 (2)	−0.019 (2)
C10	0.044 (3)	0.030 (2)	0.061 (3)	0.006 (2)	−0.019 (3)	−0.022 (2)
C11	0.048 (3)	0.052 (3)	0.068 (4)	0.006 (2)	−0.017 (3)	−0.032 (3)
C12	0.066 (4)	0.090 (5)	0.072 (4)	−0.010 (3)	−0.001 (3)	−0.044 (4)
C13	0.084 (4)	0.065 (4)	0.057 (4)	0.008 (3)	−0.008 (3)	−0.032 (3)
C14	0.088 (5)	0.093 (5)	0.080 (5)	0.013 (4)	−0.012 (4)	−0.053 (4)
C15	0.107 (6)	0.085 (5)	0.077 (5)	−0.006 (4)	0.008 (4)	−0.051 (4)
C16	0.094 (5)	0.073 (5)	0.089 (5)	0.003 (4)	−0.003 (4)	−0.045 (4)
C17	0.126 (7)	0.105 (6)	0.083 (5)	−0.001 (5)	−0.003 (5)	−0.060 (5)
C18	0.143 (8)	0.104 (6)	0.086 (6)	−0.002 (6)	0.014 (5)	−0.050 (5)

### Geometric parameters (Å, °)

**Table:**

Zn—Cl1	2.2283 (16)	C4—H4B	0.9700
Zn—Cl2	2.2626 (17)	C5—C6	1.487 (8)
Zn—N1	2.037 (4)	C5—H5B	0.9700
Zn—N4	2.026 (4)	C5—H5C	0.9700
S1—C8	1.747 (5)	C6—C7	1.518 (8)
S1—C9	1.712 (6)	C6—H6C	0.9700
S2—C10	1.721 (5)	C6—H6D	0.9700
S2—C11	1.737 (6)	C7—C8	1.504 (7)
N1—N2	1.390 (6)	C7—H7A	0.9700
N1—C9	1.339 (6)	C7—H7B	0.9700
N2—C8	1.274 (7)	C11—C12	1.502 (8)
N3—C9	1.321 (6)	C12—C13	1.495 (8)
N3—H3A	0.8600	C12—H12A	0.9700
N3—H3B	0.8600	C12—H12B	0.9700
N4—N5	1.385 (6)	C13—C14	1.511 (9)
N4—C10	1.301 (6)	C13—H13A	0.9700
N5—C11	1.273 (6)	C13—H13B	0.9700
N6—C10	1.332 (7)	C14—C15	1.530 (9)
N6—H6A	0.8600	C14—H14A	0.9700
N6—H6B	0.8600	C14—H14B	0.9700
C1—C2	1.408 (13)	C15—C16	1.514 (9)
C1—H1A	0.9600	C15—H15A	0.9700
C1—H1B	0.9600	C15—H15B	0.9700
C1—H1C	0.9600	C16—C17	1.508 (9)
C2—C3	1.548 (13)	C16—H16A	0.9700
C2—H2B	0.9700	C16—H16B	0.9700
C2—H2C	0.9700	C17—C18	1.539 (10)
C3—C4	1.525 (12)	C17—H17A	0.9700
C3—H3C	0.9700	C17—H17B	0.9700
C3—H3D	0.9700	C18—H18A	0.9600
C4—C5	1.505 (9)	C18—H18B	0.9600
C4—H4A	0.9700	C18—H18C	0.9600
			
Cl1—Zn—Cl2	114.97 (7)	C8—C7—H7A	108.6
N1—Zn—Cl1	109.00 (12)	C6—C7—H7A	108.6
N1—Zn—Cl2	106.06 (13)	C8—C7—H7B	108.6
N4—Zn—Cl1	112.65 (12)	C6—C7—H7B	108.6
N4—Zn—Cl2	108.05 (13)	H7A—C7—H7B	107.6
N4—Zn—N1	105.49 (16)	N2—C8—C7	124.3 (5)
C9—S1—C8	87.9 (2)	N2—C8—S1	113.7 (4)
C10—S2—C11	86.9 (3)	C7—C8—S1	121.9 (4)
N2—N1—Zn	115.5 (3)	N3—C9—N1	124.0 (5)
C9—N1—Zn	130.8 (4)	N3—C9—S1	123.2 (4)
C9—N1—N2	112.2 (4)	N1—C9—S1	112.8 (4)
C8—N2—N1	113.4 (4)	N4—C10—N6	124.4 (5)
C9—N3—H3A	120.0	N4—C10—S2	113.5 (4)
C9—N3—H3B	120.0	N6—C10—S2	122.1 (4)
H3A—N3—H3B	120.0	N5—C11—C12	123.8 (5)
N5—N4—Zn	115.2 (3)	N5—C11—S2	114.3 (4)
C10—N4—Zn	132.0 (4)	C12—C11—S2	121.9 (4)
C10—N4—N5	112.8 (4)	C13—C12—C11	116.6 (5)
C11—N5—N4	112.5 (4)	C13—C12—H12A	108.1
C10—N6—H6A	120.0	C11—C12—H12A	108.1
C10—N6—H6B	120.0	C13—C12—H12B	108.1
H6A—N6—H6B	120.0	C11—C12—H12B	108.1
C2—C1—H1A	109.5	H12A—C12—H12B	107.3
C2—C1—H1B	109.5	C12—C13—C14	112.7 (5)
H1A—C1—H1B	109.5	C12—C13—H13A	109.0
C2—C1—H1C	109.5	C14—C13—H13A	109.0
H1A—C1—H1C	109.5	C12—C13—H13B	109.0
H1B—C1—H1C	109.5	C14—C13—H13B	109.0
C1—C2—C3	112.5 (10)	H13A—C13—H13B	107.8
C1—C2—H2B	109.1	C13—C14—C15	113.9 (6)
C3—C2—H2B	109.1	C13—C14—H14A	108.8
C1—C2—H2C	109.1	C15—C14—H14A	108.8
C3—C2—H2C	109.1	C13—C14—H14B	108.8
H2B—C2—H2C	107.8	C15—C14—H14B	108.8
C4—C3—C2	112.1 (9)	H14A—C14—H14B	107.7
C4—C3—H3C	109.2	C16—C15—C14	114.3 (6)
C2—C3—H3C	109.2	C16—C15—H15A	108.7
C4—C3—H3D	109.2	C14—C15—H15A	108.7
C2—C3—H3D	109.2	C16—C15—H15B	108.7
H3C—C3—H3D	107.9	C14—C15—H15B	108.7
C5—C4—C3	116.1 (7)	H15A—C15—H15B	107.6
C5—C4—H4A	108.3	C17—C16—C15	113.5 (6)
C3—C4—H4A	108.3	C17—C16—H16A	108.9
C5—C4—H4B	108.3	C15—C16—H16A	108.9
C3—C4—H4B	108.3	C17—C16—H16B	108.9
H4A—C4—H4B	107.4	C15—C16—H16B	108.9
C6—C5—C4	113.5 (5)	H16A—C16—H16B	107.7
C6—C5—H5B	108.9	C16—C17—C18	113.1 (7)
C4—C5—H5B	108.9	C16—C17—H17A	109.0
C6—C5—H5C	108.9	C18—C17—H17A	109.0
C4—C5—H5C	108.9	C16—C17—H17B	109.0
H5B—C5—H5C	107.7	C18—C17—H17B	109.0
C5—C6—C7	114.8 (5)	H17A—C17—H17B	107.8
C5—C6—H6C	108.6	C17—C18—H18A	109.5
C7—C6—H6C	108.6	C17—C18—H18B	109.5
C5—C6—H6D	108.6	H18A—C18—H18B	109.5
C7—C6—H6D	108.6	C17—C18—H18C	109.5
H6C—C6—H6D	107.5	H18A—C18—H18C	109.5
C8—C7—C6	114.6 (5)	H18B—C18—H18C	109.5
			
N4—Zn—N1—C9	−143.6 (4)	C9—S1—C8—N2	−0.1 (4)
Cl1—Zn—N1—C9	−22.4 (5)	C9—S1—C8—C7	179.6 (5)
Cl2—Zn—N1—C9	102.0 (4)	N2—N1—C9—N3	−179.2 (4)
N4—Zn—N1—N2	51.9 (3)	Zn—N1—C9—N3	15.9 (8)
Cl1—Zn—N1—N2	173.1 (3)	N2—N1—C9—S1	0.3 (5)
Cl2—Zn—N1—N2	−62.5 (3)	Zn—N1—C9—S1	−164.6 (3)
C9—N1—N2—C8	−0.3 (6)	C8—S1—C9—N3	179.4 (5)
Zn—N1—N2—C8	167.0 (4)	C8—S1—C9—N1	−0.1 (4)
N1—Zn—N4—C10	−116.4 (5)	N5—N4—C10—N6	179.1 (4)
Cl1—Zn—N4—C10	124.8 (4)	Zn—N4—C10—N6	1.5 (8)
Cl2—Zn—N4—C10	−3.3 (5)	N5—N4—C10—S2	0.8 (6)
N1—Zn—N4—N5	66.0 (3)	Zn—N4—C10—S2	−176.8 (3)
Cl1—Zn—N4—N5	−52.8 (4)	C11—S2—C10—N4	−0.7 (4)
Cl2—Zn—N4—N5	179.1 (3)	C11—S2—C10—N6	−179.1 (5)
C10—N4—N5—C11	−0.4 (6)	N4—N5—C11—C12	−179.7 (5)
Zn—N4—N5—C11	177.6 (4)	N4—N5—C11—S2	−0.1 (6)
C1—C2—C3—C4	−175.8 (9)	C10—S2—C11—N5	0.5 (4)
C2—C3—C4—C5	−178.5 (7)	C10—S2—C11—C12	−179.9 (5)
C3—C4—C5—C6	178.4 (7)	N5—C11—C12—C13	−148.2 (6)
C4—C5—C6—C7	−176.8 (5)	S2—C11—C12—C13	32.2 (8)
C5—C6—C7—C8	178.3 (5)	C11—C12—C13—C14	175.9 (6)
N1—N2—C8—C7	−179.5 (5)	C12—C13—C14—C15	−178.1 (6)
N1—N2—C8—S1	0.2 (6)	C13—C14—C15—C16	175.3 (6)
C6—C7—C8—N2	178.9 (5)	C14—C15—C16—C17	−179.8 (7)
C6—C7—C8—S1	−0.8 (7)	C15—C16—C17—C18	177.3 (7)

### Hydrogen-bond geometry (Å, °)

**Table:**

*D*—H···*A*	*D*—H	H···*A*	*D*···*A*	*D*—H···*A*
N3—H3A···Cl1	0.86	2.58	3.374 (5)	154
N3—H3B···Cl2^i^	0.86	2.77	3.503 (5)	144
N6—H6A···Cl2	0.86	2.49	3.289 (5)	155
N6—H6B···N2^ii^	0.86	2.19	3.018 (3)	163

Symmetry codes: (i) −*x*, −*y*+2, −*z*+1; (ii) −*x*, −*y*+1, −*z*+1.
